# Welfare assessment of dromedary camels kept under pastoralism in Pakistan

**DOI:** 10.3389/fvets.2024.1442628

**Published:** 2024-10-30

**Authors:** Barbara Padalino, Asim Faraz, Naod Thomas Masebo, Abdul Waheed, Hafiz Muhammad Ishaq, Nasir Ali Tauqir, Ali Raza Abbasi, Laura Menchetti

**Affiliations:** ^1^Department of Agricultural and Food Sciences, University of Bologna, Bologna, Italy; ^2^Faculty of Science and Engineering, Southern Cross University, Lismore, NSW, Australia; ^3^Department of Livestock and Poultry Production, Bahauddin Zakariya University, Multan, Pakistan; ^4^School of Veterinary Medicine, Wolaita Sodo University, Wolaita Sodo, Ethiopia; ^5^Department of Animal Nutrition, The Islamia University of Bahawalpur, Bahawalpur, Pakistan; ^6^Faculty of Veterinary and Animal Sciences, MNS University of Agriculture, Multan, Pakistan; ^7^School of Biosciences and Veterinary Medicine, University of Camerino, Camerino, Italy

**Keywords:** animal-level, camels, Good feeding, Good housing, Good health, Good behavior, herd-level

## Abstract

Standardized welfare assessment protocols are crucial to enhance animal welfare; up to date, there is no data on the level of welfare of camels kept under pastoralism. A tailored protocol for measuring welfare in dromedary camels kept under nomadic pastoralist conditions was recently developed, drawing from the currently available welfare protocol for dromedary camels kept in intensive systems. This study, therefore, aimed to apply the newly developed tailored protocol and assess the welfare of dromedary camels kept under pastoralism in the Southern Punjab Province of Pakistan. A total of 44 welfare indicators (animal-, resource, and management-based measures) aligning with animal welfare principles (“Good Feeding”, “Good Housing”, “Good Health”, and “Appropriate Behavior”) were gathered into two assessment levels: “Caretaker-Herd level” and “Animal level”. Data were collected in 2023 in the Cholistan desert in the southern Punjab province. Fifty-four herds were evaluated for a total population of 1,186 camels, of which 510 (495 females and 15 males; average age: 5–6 years old) were assessed at the animal level. The indicators were scored and aggregated to obtain Principle Aggregated Indexes (PAIs) and a total Welfare Index (TWI). Using the PAIs classification, 4 herds were categorized as excellent, 42 satisfactory, and 8 unsatisfactory. Total Welfare Index (TWI) varied from 55.7 to 82.2, and the thresholds for classification into tertiles were 65.4 and 70.6. Good feeding and Good housing were the most problematic PAIs, with Good feeding as the most influential variable for classification into welfare categories. As expected, camels kept under pastoralism had a higher level of welfare than those reported in the literature for intensive systems, especially concerning the Appropriate Behavior principle. Our findings are a first step in proposing welfare standards for dromedary in Pakistan and worldwide.

## 1 Introduction

Assessing animal welfare requires a multidimensional approach which may vary depending on the husbandry system (1, 2). Assessing the welfare of livestock in extensive systems requires a comprehensive understanding of the heterogeneous extensive environment, the behavior of the animal, human-animal relationships (stockmanship), health factors, and observing the animals during routine husbandry practices (2). Dromedaries or one-humped camels (*Camelus dromedarius*) have long been integral to the livelihoods and cultural identity of many societies across arid and semi-arid regions of the world (3, 4). Renowned for their adaptability to harsh environmental conditions, dromedaries are mostly prized for their utility in transportation, milk production, and meat provision (3). Their significance extends beyond mere economic considerations, as they often serve as symbols of resilience and endurance in the face of adversity (5). Currently, in different regions of the globe, there’s a growing body of research focused on milk quality, production, health, reproduction, nutrition, calf management, and management systems of dromedary camel. However, there remains a notable scarcity of research addressing the welfare of dromedary camels in diverse management systems (6).

The global landscape of dromedary camel rearing and production is undergoing significant transformation, driven by varying socio-economic interests and evolving husbandry practices. Increasingly, dromedaries are integrating into intensive and semi-intensive production systems, reflecting shifts in consumer demand and agricultural trends (7, 8). Recognizing the inherent relationship between animal welfare and production outcomes (i.e., improved productivity and product quality), efforts have been made to develop assessment tools for evaluating the welfare of dromedary camels within intensive and semi-intensive regimes (9). However, it is important to note that the vast majority of the global dromedary population is raised under nomadic, traditional pastoralist conditions in the arid and semi-arid ecosystems of Africa and Asia (4, 10). Nomadic pastoralism represents a traditional husbandry system where livestock are allowed to graze freely over vast expanses of rangeland. This lifestyle is intimately intertwined with the ecological and socio-cultural dynamics of arid regions, where sedentary agriculture may be impractical (11).

Despite the prevalence of nomadic pastoralism in dromedary camel husbandry, animal welfare assessment in these extensive production systems has received limited attention in research (6, 12). This discrepancy is partly attributable to the perception that animals in open ranges enjoy natural lives, thus minimizing welfare risks (13). While it is true that animals at pasture can exhibit a broader range of behaviors and enjoy environmental enrichment, the challenges associated with outdoor living, such as thermoregulation, the presence of predators, and nutritional inadequacy, necessitate careful consideration (2, 14). Moreover, the traditional knowledge and practices governing camel husbandry may not always align with modern standards of animal welfare (15). In response to these challenges, a new protocol tailored specifically to the unique conditions of dromedary camels reared under nomadic pastoralist conditions has recently been proposed but has not yet been applied (16). Within the Asian continent, Pakistan emerges as a prominent hub of dromedary camel pastoralism (17, 18). Despite the cultural and economic significance of dromedaries in this country, there remains a dearth of scientific research elucidating the welfare implications associated with nomadic pastoralist systems. Understanding the welfare challenges and opportunities inherent in these production systems is crucial for devising targeted interventions aimed at improving the lives of both animals and their human caretakers. Moreover, such insights can apprise policy decisions aimed at promoting sustainable and ethically sound livestock production practices. We hypothesized that dromedary camels under pastoralism could live in their natural environment and express natural behaviors, having an acceptable welfare level, but with the applied protocol, we could identify some challenges, such as limited feed and water due to environmental conditions.

In light of these considerations, employing the newly developed welfare assessment protocol tailored to this specific context (16), this study aimed to assess the welfare status of dromedary camels kept under pastoralism in the Southern Punjab Province of Pakistan.

## 2 Materials and methods

This study was approved by the Bahauddin Zakariya University Animal Ethics Committee (Protocol number DLPP/272/27-11-2023). A native speaker research team member (ARA) facilitated the meeting between the assessor and the herd manager or owner. During this meeting, the objectives and methods of the welfare protocol were explained, and permission to carry out the assessment protocol was obtained.

### 2.1 Study location and sampling

**Figure 1:**
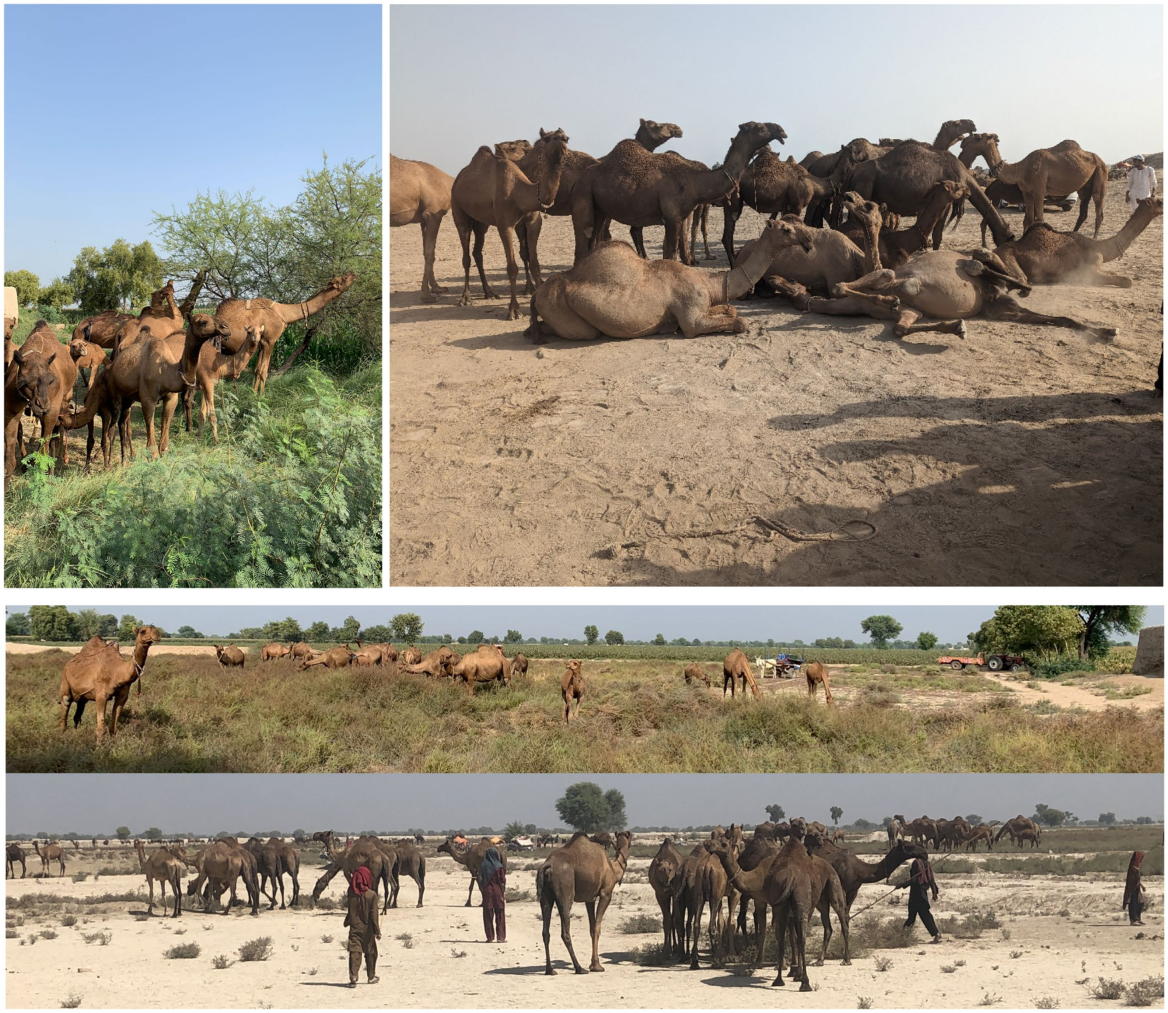
Examples of different camel herds assessed in different locations.

A prospective field study was performed in the southern Punjab province, Pakistan, from 29th September to the 7th October 2023. Mean ambient temperature and relative humidity were 33.4°C (min-max: 21.3°C–40.9°C) and 48.1% (min-max: 20.9–88%), respectively. Fifty-four dromedary camel pastoralist herds inhabiting eight different localities within the neighborhood of Multan and the Cholistan desert (Lohari Gate (*n* = 11), Kotla Pul (*n* = 10), Channan Pir (*n* = 9), Gelewala (*n* = 7), Nag Shah (*n* = 5), Naubahar Pul (*n* = 5), Gograan (*n* = 4), and Chak 97 (*n* = 3)) were visited for welfare assessment (Figure 1).

One of the authors (Dr Ali Raza Abbasi) contacted local community leaders to facilitate and ease the communication with herd managers/ pastoralists. Subsequently, herd managers/ pastoralists were approached in collaboration with these leaders and their networks, ensuring their participation remained entirely voluntary. According to the previous schedule, each herd was visited only once to respect pastoralists’ routines. Herd size ranged from 5 to 63 animals, with an average size of 43 individuals. The most popular breeds in the area were Barela and Marecha. The management practices across all herds were broadly similar. The camels were permitted to roam and feed in the nearby cultivated areas throughout most of the day and sometimes provided supplemental feed sourced from grass collected from agricultural fields. Milk production and camel rearing for sacrificial purposes were the primary breeding purposes of all the herds involved in the present study. Females were milked twice daily (morning and afternoon), with an average milk production of 9.5 liters/day. The collected milk was partly used for household and sold out to urban vendors to help generate income for the pastoralists. Most male calves were usually sold around 1 year of age during fairs/festivals, mainly for meat production (sacrificial purposes). Following the protocol (16), only adult animals (>3 years old) were assessed.

At each herd, the number of dromedary camels for assessment at the Animal level was selected randomly following the general statistical rules reported by Padalino and Menchetti (9). As required by the protocol, animals under treatment for severe or acute pathological conditions such as chronic and acute mastitis, abscess, chronic wounds, and chronic skin infections were excluded from this calculation (Supplementary Figure S1).

In total, 510 adult dromedary camels (495 females and 15 males; average age: 5–6 years old) were assessed. Based on physiological status, females were differentiated into 3 different categories: lactating (*n* = 234), pregnant (*n* = 162), and non-lactating/non-pregnant (*n* = 99). It is worth mentioning that females were naturally mated, and pregnancy diagnosis was carried out by observing an erect and coiled tail in the pregnant animal when approached by a male camel (“tail cocking”) (19). As the pregnancy status could not double-checked using ultrasonography, the previous classification could be inaccurate, and it was not further used. None of the assessed herds practiced male castration. Few herds had a breeding male within the herd, but most used to introduce a breeding bull during the reproductive season. These breeding male were kept in different locations and were not assessed in the current study.

### 2.2 Welfare assessment protocol

The welfare assessment was performed following the protocol by Padalino and Menchetti (16). It contained 44 welfare indicators (animal, resource, and management-based measures) aligning with animal welfare principles (“Good Feeding,” “Good Housing,” “Good Health,” and “Appropriate Behavior”). Indicators were gathered into two assessment levels: “Caretaker-Herd level” and “Animal level”. The assessment at the caretaker herd level was performed by the native speaker research team members (FA, TNA), while the Animal level was conducted by the lead researcher (BP). A three-point scale was used to score the indicators: 0 denoting good welfare, 1 indicating compromised welfare, and 2 reflecting unacceptable welfare. For indicators with binary responses (e.g., presence/ absence), only scores of 0 (good welfare) and 2 (unacceptable welfare) were taken into account.

The “Caretaker-Herd level” consists of a face-to-face interview with the herd manager or owner. This interview included 16 unique closed-ended questions that probed various aspects such as feeding and healthcare practices, housing conditions, and the handling experience. At the end of the interview, the assessors watched from a distance as caretakers (those who handle, feed, and water the animals) interacted with the camels. The caretakers’ mood and techniques were observed, as well as the use of any equipment (such as a stick) and how it was used, and this observation was taken into consideration to score the caretaker’s attitudes toward animal handling.

The “Animal level” included 27 distinct welfare-related indicators that always aligned with the four welfare principles. The selected indicators were applied to assess feeding and watering practices, thermal and resting comfort, monitor health conditions, and observe the behavior of the animals in relation to the environment, their conspecific, and humans. At this assessment level, after randomly picking the camels to be assessed, a 3-min behavioral observation was carried out without disturbing the animal. This observation records parameters for “Good Housing” (such as access to shaded places, risk of harm, and voluntary resting behavior), “Good Feeding” (including food and water availability), and “Appropriate Behavior” principles. The “Appropriate Behavior” principle entails an approaching test as in previous studies (12, 20). Briefly, the assessor approaches the camel slowly from the side, extending their arm and hand in a non-threatening manner. The test was completed if the camel demonstrated avoidance or hostile behavior or if the assessor successfully approached and placed a hand near the camel’s nose. The camel’s response could be negative, neutral, or positive, representing protective or anxious behavior, calmness, or interest and involvement, respectively.

After the behavioral test, a visual inspection was performed to determine the camel’s Body Condition Score (BCS) and other indicators of “Good Housing” (such as the presence of ectoparasites, cleanliness, and physical restraint), as well as to look for clinical signs, pain-induced practices, and injuries listed in the “Good Health” principle. The camel’s ribs, ischial and coxal tuberosities, flank hollow, and recto-genital zone are used to assess body condition on a 0–5 scale (21). The presence of bleeding and open wounds was also noticed, with bleeding denoting obvious blood flow and open wounds, including damaged skin that exposes underlying tissues. Furthermore, if a dromedary camel remained resting during the assessment, it was gently coaxed to stand and move a few steps to determine lameness. This enabled the evaluation of the camel’s gait to establish whether it could carry weight evenly and if there were any interruptions in its movement. In contrast, if the camel needed assistance to stand or could not bear weight on one leg, or exhibited a relieving posture, assessing its gait in motion was not necessary to confirm lameness. Assessed camels were marked to avoid reassessment, as freely moving camels can be challenging to track. The welfare assessment data were documented manually on paper utilizing the protocol’s example recording sheets (16). Once fieldwork was completed, data were transferred into an Excel recording sheet for further analysis.

### 2.3 Calculation of partial and aggregate welfare indices

The indices of welfare were calculated following Menchetti et al. (22) and as reported in full in Padalino and Menchetti (12). Briefly, the measures’ scores were initially summed into 8 partial indices (PIs) corresponding to the 4 welfare principles (“Good Feeding,” “Good Housing,” “Good Health,” and “Appropriate Behavior”) evaluated at the two levels (“Caretaker-Herd” and “Animal level”). In these PIs, the initial 0–2 scale was translated into a 0–100 scale, with 0 representing the lowest (i.e., unacceptable welfare) value and 100 representing the highest (i.e., optimal welfare).

Subsequently, PIs were merged into weighted sums, resulting in aggregated indices for each welfare principle, known as Principle Aggregate Indices (PAIs; Good Feeding Index, Good Housing Index, Good Health Index, and Appropriate Behavior Index). In the PAIs, a lower weight (20%) was assigned to the PAIs obtained at the “Caretaker-Herd level” as it mainly includes indicators self-reported by the farmer from memory, hence susceptible to potential “questionnaire bias”. Finally, the Total welfare index (TWI) was calculated as a weighted sum of the 4 PAIs (12, 22).

### 2.4 Classification of herds

Various welfare classes were established to categorize the herds based on the four PAIs (i.e., “welfare profiles system”). Each herd was classified using a mixed rule system that involved comparing the PAI scores of the herd with predefined reference profiles. According to Menchetti et al. (22), herds were classified as “Excellent” if all PAIs were over 60 and two were over 80; “Satisfactory” if all PAIs were over 30 and three were over 60; “Unsatisfactory” if all PAIs were over 20 and three were over 30; and “Unacceptable” if they failed to meet the abovementioned criteria.

Furthermore, an additional type of classification was used (22) based on the tertiles of the TWI (i.e., “traffic light system”); herds could thus have a “green light” if their TWI belongs into the third tertiles, an “orange light” if the TWI belongs to the second tertiles, or a “red light” if their TWI belongs to the first tertiles.

### 2.5 Statistical analysis

Descriptive statistics was performed and data presented as absolute and relative frequencies, median (Mdn), maximum (Max) and minimum (Min) values, first and third quartiles, and interquartile range (IQR). For the indicators at the “Caretaker-Herd level”, frequencies were presented relative to the total number of assessed herds (*n* = 54). At “Animal-level”, frequencies were expressed in relation to the total number of assessed dromedary camels (*n* = 510). One sample binomial or Chi-square tests were used to compare the observed distributions with the expected probability distributions (each assuming all categories equal). Differences between medians of partial or aggregated indices were analyzed using related-samples Friedman tests with Bonferroni correction for multiple comparisons, while Wilcoxon signed tests were used to compare the scores obtained at Caretaker-Herd and Animal levels. Finally, discriminant analyses (DAs) were used to define the relative importance of each welfare principle in classifying the herds. In the DAs, the welfare categories (i.e., Excellent, Satisfactory, and Unsatisfactory for the “welfare profiles system”; Green, Orange, and Red Light for the “traffic light system”) were included as grouping variables while the four PAIs scores were included as independent variables. The standardized coefficients of the discriminant functions indicated the relative importance of each PAI in classifying the herds. The centroids (i.e., mean discriminant scores of the discriminant functions) were used to establish the cutting point for classifying herds (23, 24). Statistical analysis was performed using SPSS Statistics for Windows, version 25.0, IBM Corp. (2017), while GraphPad Prism, version 7.0 (GraphPad Software, San Diego, CA, United States) was used for the data visualization. *p* values <0.05 were considered statistically significant.

## 3 Results

### 3.1 Welfare assessment at the Caretaker-Herd level

The scores obtained from 54 caretakers-herd level assessments were presented in Table 1. The majority of caretakers, 36/54 (66.7%; *p* < 0.001), depend only on grazing for feeding their animals, while only a small fraction, 10/54 (18.5%), stated they provided supplementation in addition to grazing. All caretakers said their animals had access to water more than once a day. However, most caretakers 47/54 (87%; *p* < 0.001) revealed that their camels lacked shaded areas, and 21/54 (38.9%; *p* = 0.134) stated their camels lacked overnight resting places. Although all caretakers claimed their animals’ received vaccinations, only a few 6/54 (11.1%; *p* < 0.001) stated they used a veterinarian to treat sick animals. In terms of experience, all caretakers had over 10 years of camel rearing experience, with a significant portion 42/54 (77.8%; *p* < 0.001) showing gentle handling practices with their camels.

**Table 1:** Frequency table of the scores of the welfare indicators obtained at the Caretaker-Herd level corresponding to the four welfare principles (i.e., Good feeding, Good housing, Good health, and Appropriate behavior) collected from 54 herd caretakers/owners in Pakistan in 2023.

Welfare principle	Question/welfare indicator	Answer/observation	Scoring scale	Number	Percentage	*p* value
Good feeding	How often do you feed the camels?	Grazing for around 10–12 h per day + supplementation	0	10	18.5	<0.001
		Only grazing for 10–12 h per day	1	36	66.7	
		Only grazing for less than 6–8 h per day	2	8	14.8	
	How often do you water the camels?	Always available	0	0	0	–
		Available more than once daily	1	54	100	
		Available less than once daily	2	0	0	
Good housing	Do camels have a resting place overnight?	Yes	0	21	38.9	0.134
		No	2	33	61.1	
	How many adult animals do you have in your herd?	<30 camels (*Small size*)	0	46	85.2	<0.001
		>30 camels (*Large size*)	2	8	14.8	
	Do the camels have access to shaded areas?	Free access during the whole day	0	0	0	<0.001
		For a short time period of time per day	1	7	13	
		Never	2	47	87	
	Do you practice any type of predator control?	Yes	0	54	100	–
		No	2	0	0	
Good health	Who routinely assess the camel’s health?	A veterinarian	0	6	11.1	<0.001
		A non-veterinarian	1	48	88.9	
		Not conducted	2	0	0	
	Who treats the camels when they are sick?	A veterinarian	0	6	11.1	<0.001
		A non-veterinarian	1	48	88.9	
		Not conducted	2	0	0	
	Are vaccinations routinely conducted?	Yes	0	54	100	–
		No	2	0	0	
	Is deworming routinely conducted?	Yes	0	6	11.1	<0.001
		No	2	48	88.9	
	What is the 1-year-old calf mortality rate?	Below 10%	0	0	0	–
		Over 10%	1	0	0	
		Records not available	2	54	100	
	Do you identify your animals?	Yes, using non-invasive methods	0	21	38.9	<0.001
		Yes, using pain-induced practices	1	33	61.1	
		No	2	0	0	
	Do your animals have the possibility to have contact with other livestock herds (commingling)?	No	0	0	0	–
		Rarely	1	0	0	
		Yes	2	54	100	
Appropriate behavior	Do you have any aggressive/dangerous animals in your herd?	No	0	32	59.3	<0.001
		Yes, but only during the breeding season	1	0	0	
		Yes	2	22	40.7	
	How many years of experience in handling camels do you have?	More than 10	0	54	100	–
		Between 5 and 10	1	0	0	
		< 5 years	2	0	0	
	What is the ratio between the number of caretakers and the number of animals kept in the herd?	Ratio ≥ 0.05 (1/20)	0	54	100	–
		Ratio < 0.05 (1/20)	2	0	0	
	Caretaker attitudes during animal handling	Speaks, touches and/or whistles softly/quietly	0	42	77.8	<0.001
		Speaks, touches and/or whistles harshly/loudly	1	6	11.1	
		Speaking/shouting impatiently, forceful use of stick/hand	2	6	11.1	

– means that the *p* value was not calculated as the distribution of the answers was constant.

### 3.2 Welfare assessment at the animal level

The assessment findings of the 510 dromedary camels assessed at the Animal level are summarized in Table 2. Regarding the Good feeding principle, 69% (352/510) of animals had access to food, but only 89/510 (17.5%) had access to water during the inspection (*p* < 0.001; Supplementary Figures S2A,B). Body condition scores (BSC) evaluation indicated that 267/510 (52.4%) had moderate and 35.3% (180/510) had good body conditions (*p* < 0.001; Supplementary Figure S2C). Concerning the Good housing principle, 469/510 (92%) camels have no shade available at the time of evaluation (*p* < 0.001; Supplementary Figure S2D). The most frequent health issues detected during the evaluation were skin disorders, 181/510 (35.5%), discharge 140/510 (27.5%), and injuries 51/510 (10%) (Supplementary Figures S2E,F). Despite overall good health, 152/510 (29.8%) of the camels had experienced pain-induced management practices (i.e., cauterization, branding, nose pag, mutilation; Supplementary Figure S2G). Behavioral observations revealed a minimal incidence of animals showing stereotypy (2/510; 0.2%) and aggressive interactions (10/510; 2%). At the approaching test, the majority of the camels showed a positive response (272/510, 53.3%), while a third of them (151/510, 29.6%) were neutral, and only 87/510 (17.1%) showed negative interactions (*p* < 0.001; Supplementary Figure S2H).

**Table 2:** Frequency table of the scores of the welfare indicators obtained at the animal level corresponding to the four welfare principles (i.e., Good feeding, Good housing, Good health and Appropriate behavior) collected from 510 camels in Pakistan in 2023.

Welfare principle	Welfare indicator	Observation	Scoring scale	Number	Percentage	*p* value
Good feeding	Food availability	Yes	0	352	69	<0.001
		No	2	158	31	
	Water availability	Yes	0	89	17.5	<0.001
		No	2	421	82.5	
	Body condition score	BCS = 3 (good body condition)	0	180	35.3	<0.001
		BCS = 2 or BCS = 4 (moderate body condition)	1	267	52.4	
		BCS = 0–1 or BCS = 5 (cachexia or obesity)	2	63	12.4	
Good housing	Currently available shade	Yes	0	41	8	<0.001
		No	2	469	92	
	Risk of injury	No	0	445	87.3	<0.001
		Yes	2	65	12.7	
	Presence of ectoparasites	No	0	391	76.7	<0.001
		Yes	2	119	23.3	
	Camel coat cleanliness	Clean	0	375	73.5	<0.001
		Partially clean	1	112	22	
		Dirty	2	23	4.5	
	Tethered	No	0	488	95.7	<0.001
		Yes	2	22	4.3	
	Restrained into two/three legs	No	0	365	71.6	<0.001
		Yes	2	145	28.4	
	Hobbled	No	0	486	95.3	<0.001
		Yes	2	24	4.7	
	Voluntary resting behavior	Yes	0	143	28	<0.001
		No	2	367	72	
Good health	Presence of bleeding	No	0	477	93.5	<0.001
		Yes	2	33	6.5	
	Presence of injury (open wounds)	No	0	459	90	<0.001
		Yes	2	51	10	
	Presence of swollen joints	No	0	495	97.1	<0.001
		Yes	2	15	2.9	
	Presence of lameness	No	0	507	99.4	<0.001
		Yes	2	3	0.6	
	Presence of skin disorders	No	0	329	64.5	<0.001
		Yes	2	181	35.5	
	Presence of discharge (nose, eye, vulva)	No	0	370	72.5	<0.001
		Yes	2	140	27.5	
	Presence of diarrhoea	No	0	499	97.8	<0.001
		Yes	2	11	2.2	
	Presence of respiratory disorders	No	0	507	99.4	<0.001
		Yes	2	3	0.6	
	Presence of other health disorders	No	0	425	83.3	<0.001
		Yes	2	85	16.7	
	Presence of pain-induced management practices (cauterization, branding, nose pag, mutilation)	No	0	358	70.2	<0.001
		Yes	2	152	29.8	
	Evident pain	No	0	493	96.7	<0.001
		Yes	2	17	3.3	
Appropriate behavior	Positive social camel-camel interactions (cow-calf contact, allogrooming, sniffing)	Yes	0	339	66.5	<0.001
		No	2	171	33.5	
	Aggressive camel-camel interactions	No	0	500	98	<0.001
		Yes	2	10	2	
	Stereotypies	No	0	508	99.6	<0.001
		Yes	2	2	0.4	
	Feeding or rumination	Yes	0	207	40.6	<0.001
		No	2	303	59.4	
	Approaching test	Positive	0	272	53.3	<0.001
		Neutral	1	151	29.6	
		Negative	2	87	17.1	

### 3.3 Partial and aggregate welfare indices

**Figure 2:**
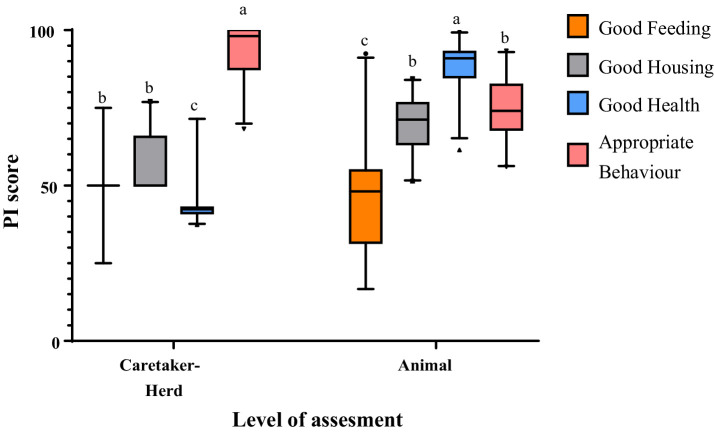
Boxplot for partial indices (PIs) at Caretaker-Herd and Animal levels corresponding to the four welfare principles (i.e., Good feeding, Good housing, Good health, and Appropriate Behavior). The whiskers on the plot define the range from the 2.5 to the 97.5 percentile, while the dots show the outliers (that fall below or above this range). Boxes not sharing any superscript (a,b,c) for each level of assessment are significantly different at *p* < 0.05.

The partial indices (PIs) statistics are presented in Figure 2 and Supplementary Tables S1, S2. At the Caretaker-Herd level, the partial indices (PIs) of Appropriate Behavior had the highest median, followed by Good Feeding and Good Housing whereas Good Health obtained the lowest median (*p* < 0.001). At the Animal level, the highest score was found for Good Health, followed by Appropriate Behavior and Good housing, while Good feeding obtained the lowest median (*p* < 0.001).

The comparison between assessment levels (Supplementary Figure S3) showed that the PIs of Good Housing and Good Health were higher at the Animal level than at the Caretaker-Herd level, while the PI of Appropriate Behavior was higher at the Caretaker-Herd level (both *p* < 0.001). No difference was found instead for the Good Feeding principle (*p* = 0.423).

**Figure 3:**
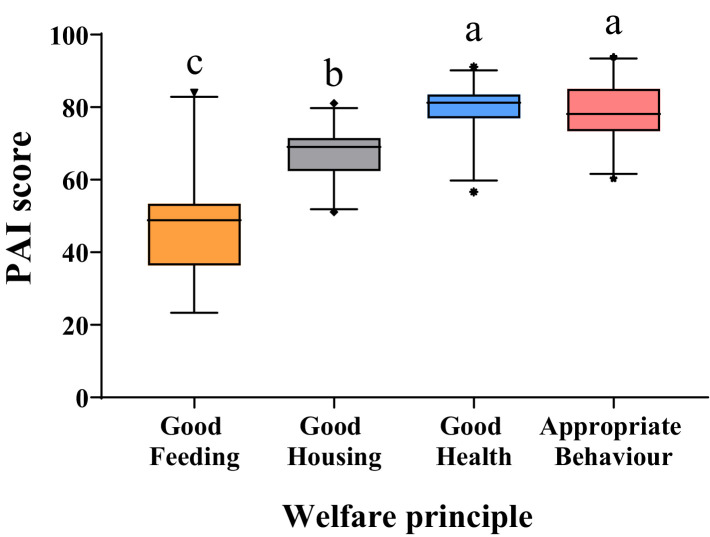
General boxplot for partial aggregate indices (PAIs) corresponding to the four welfare principles. The whiskers on the plot define the range from the 2.5 to the 97.5 percentile while the dots show the outliers (that fall below or above this range). Boxes not sharing any superscript (a,b,c) are significantly different at *p* < 0.05.

The statistics of the principal aggregate indices (PAIs) corresponding to the four welfare principles are shown in Figure 3. The PAIs of Good health and Appropriate behavior had the highest median, followed by Good Housing and finally by Good feeding (*p* < 0.001). The Good feeding principle also showed the greatest variability between herds (range: 23.33–83.94).

### 3.4 Classification of herds

The herds were classified into welfare categories based on the computed PAIs (“welfare profiles system”) and TWI (“traffic light system”). The classifications of each herd based on PAIs were presented in Supplementary Table S3. In summary, four herds obtained excellent, forty-two satisfactory, eight unsatisfactory and none of the herds obtained unacceptable category (Table 3). Furthermore, using TWI, the herds were categorized into three tertiles, and the classifications of each herd based on TWI were presented in Supplementary Table S4. Total Welfare Index (TWI) ranged from 55.7 to 82.2. Herds (*n* = 12) that fell into the third tertile (i.e., green light) had a TWI score > 70.6, those in the second tertile (i.e., orange light; *n* = 22) had a score between 65.4 and 70.6, while those in the first tertile (i.e., red light: *n* = 20) had a score ≤ 65.3.

**Table 3:** The summary of the classification of the welfare category of the herds based on the principal aggregate indices (PAIs; “welfare profiles system”).

Welfare category	Criteria	Number of herds
Excellent	>60 for each PAI and > 80 for at least two PAIs	4
Satisfactory	>30 for each PAI and > 60 for at least three PAIs	42
Unsatisfactory	>20 for each PAI and > 30 for at least three PAIs	8
Unacceptable	Failure to meet the abovementioned criteria	0

**Figure 4:**
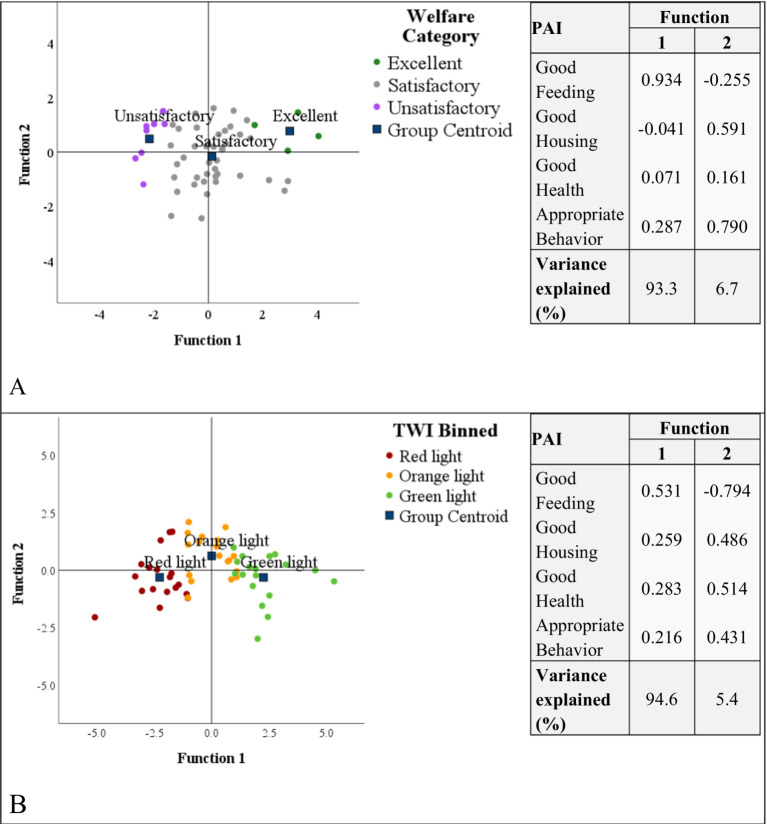
Scatterplots of discriminant scores for Function 1 vs. Function 2 and structure matrix indicating the standardized coefficients of the discriminant functions. (A) Presents the results of the discriminant analysis of the “welfare profiles system,” while (B) presents the results of the discriminant analysis of the “light traffic system” (B).

Figure 4 shows the discriminant scores of the DAs carried out to identify the most important variables in the classification according to the “welfare profiles system” (Panel A) and “light traffic system” (Panel B). The first functions extracted explained more than 90% of the variance for both systems, and it had the highest coefficients for the Good Feeding variable. This suggests that this PAI was the most influential variable in classifying herds. The coefficients of the Good Feeding variable were positive, as well as the centroids for both the Excellent and Green light categories (Supplementary Table S5), indicating that herds having high scores for Good Feeding PAI could achieve the highest levels of welfare.

## 4 Discussion

The present study employed a recently developed protocol to assess the welfare of dromedary camels under nomadic pastoralist conditions (16), adapted from existing welfare protocols for dromedary camels in intensive and semi-intensive systems (9). Our study facilitated a better understanding and recognition of key welfare concerns among dromedary camels under pastoralism in Pakistan. Through this initiative, our goal was not only to enhance scientific knowledge but also to foster informed decision-making and policy formulation, promoting sustainable and ethical management of dromedary camel populations in Pakistan and globally. To the best of our knowledge, this could be the first welfare assessment of dromedary camels managed under a nomadic pastoralist production system using a standardized protocol (16), which also reports the classification of the herds. The application of a standard protocol allows the possibility to compare different herds and animals kept in different countries, develop benchmarking, and propose minimal welfare standards.

The initial stage of our assessment targeted the caretakers or herd keepers, who typically were the owners of the camels in most of the cases in Pakistan. This was expected as camel breeding under pastoralism generally and in this country is mainly based on the family tradition; each kid heredities one or more camels, and then he/she can start his/her own camel herd when grows up (25, 26). Caretakers primarily play an important role in caring, treating, and gently handling their animals; their experience and awareness are important factors as they may lead to fear and other negative emotions in the managed animals (27), and information gathered from them helps to determine the welfare status of their animals (9, 27). The pastoralists/ herders in the study area, specifically the “Cholistan” desert, move their animals seasonally in search of grazing pasture and water for their animals (18). The majority of the caretakers indicated grazing as the sole feeding practice. The availability of grazing vegetation in pastoral areas depends on the amount of rainfall, and it may become a welfare concern. There are no problems when the rainfall is high (more than 250 mm or more), as it results in expansive grazing areas covered by vegetation for the animals (28), but during dry periods, this is not the case. Moreover, Raziq et al. (18) stated that pastoralists faced a shortage in grazing land due to the increased mechanized land cultivation for wheat and cotton production in the study area. As a matter of fact, the Good Feeding score at the Caretaker-Herd level showed considerable variability between herds, suggesting that the supply of feed and water is highly variable depending on their availability in the environment and the keeper. Even though the herd caretakers claimed that their animals get water more than once a day, the availability of water depends on the season, and the available source of water in the area is primarily ponds, either natural or man-made, called “Tobas” and underground water (17, 18, 28). In agreement with our findings, Faraz et al. (17) reported that herders claimed they provide water 2–3 times a day to their camels. Appropriate feeding and watering practices are also important for production and reproduction; indeed, puberty depends on the skeletal maturity and body condition score (BCS) of the animals (29, 30). Integrating the diet when the pasture quality is low may be a best practice not only for the welfare of the animals but also to enhance sexual maturity and start breeding careers earlier.

Almost all camels lacked shaded areas during the assessment, and almost half of the caretakers stated their camels had no overnight resting places. Our findings are in line with the literature (31). Faraz et al. (17) reported similar findings for other areas of Pakistan where most camels were reared in open and semi-open facilities. However, regarding shade, Faraz et al. (17) reported that the camels were managed with the availability of many trees during hot days, and herders made semi-open shade using bamboos and “Sirki.” This disagrees with our findings, as in most cases, there were no trees or “Sirki” during our assessments. Furthermore, the literature has tested that ensuring livestock resting areas at night serves crucial purposes, safeguarding them from potential dangers such as predator attacks and protecting them from extreme environmental stressors (4). While conducting our research, we observed a few herds at an assembly point called “Channan Pir rest house.” This assembly/resting point was constructed by the government. It had water facilities, water tanks, and shaded resting areas for camels and animals, and it was an ideal place where animals could be fenced overnight. Similar 50 watering points were also constructed in the desert area of Cholistan. This initiative should be replicated and extended to other isolated areas to improve the well-being of the camels.

According to most caretakers, a non-veterinarian treated sick camels. The lack of veterinary structure and services in the far-flung area of the desert, coupled with the lack of trust by pastoralists in the service provided by the veterinarian, leads the pastoralists to prefer traditional practices and ethnoveterinary medicine to treat their animals (18). In fact, in a study in Qatar (32) and Egypt (33) camel markets, caretakers treat and self-administer the camels. Faraz et al. (17) reported herders use traditional and ethnoveterinary medicine to treat their animals in the Bhakkar district in the province of Punjab, Pakistan. In many pastoralist and camel-rearing communities, the widespread practice of traditional and ethnoveterinary medicine for treating camels is evident (17, 34–36). While the use of traditional and ethnoveterinary medicines has some potential benefits, it also has several risks to the health and welfare of the camels. Therefore, strategies to improve the pastoralists’ attitude toward using modern veterinary services should be realized. A detailed investigation of the indigenous traditional practices and ethnoveterinary medicine knowledge should be conducted to improve and use it in a better way that does not degrade the welfare of the camels. Moreover, the lack of animal identification methods and proper record-keeping of health could pose a challenge for pastoralists, making it difficult to enhance the overall health status of the camels. In many countries, camel identification is not mandatory, which impairs any possible disease prevention/eradication. Many camels around the world are moved from area to area and from country to country. All those movements should be recorded to minimize the risk of spreading infectious diseases. A campaign on camel identification for proper tracking of the animal is suggested not only to improve camel health and welfare but also human safety. The limited recourse to veterinary care, the use of pain-induced management practices, and the lack of health monitoring and record practices can explain the low score of the partial index (PI) of Good Health at the Caretaker-Herd level. A similar finding has been reported by Lamuka et al. (34) in Kenya, where pastoralist use of veterinary services is very low, and absence of treatment or health records of camels. Globally, in pastoralist communities, the use of modern veterinary services is scarce due to low availability and also low awareness of the use of modern veterinary services (18, 37). In addition, the distribution of diseases is high in most pastoral management systems, making camel production challenging by affecting their production and well-being (38–40). Stakeholders bear a great responsibility in implementing necessary policies to improve and facilitate the availability of veterinary services and provide adequate training to instill confidence in pastoralists in modern veterinary services. In addition, the government needs to ensure the availability of veterinarians and community animal health workers who are selected from the community, and train and work within the community. This approach will help to enhance the accessibility of veterinary services within the communities and the superior welfare of the camel.

In the current study, most caretakers had long experience in camel handling, and they used to treat their animals gently. The good caretaker-camel relationship was also supported by the results of the approaching tests, during which most camels responded positively or neutrally. Herders’ experience and knowledge in appropriate handling greatly impact the welfare and health of camels (36). The principle of Appropriate behavior, in fact, received a good score at both the Caretaker-Herd and Animal levels. Our data have, therefore, supported the findings of previous researchers, as it was found that the experience in handling and care provided by caretakers and herd keepers significantly influenced the response to the approaching test (41). Education on animal handling based on learning theory has been suggested to improve animal welfare in other species (42, 43); increasing knowledge on animal behavior and how to assess animal welfare have been proven to be useful in enhancing animal welfare (33). Thus, workshops on these topics should be implemented in countries where the cruel handling of camels and poor knowledge of animal welfare have been reported in the media (44).

The assessment at the Animal level was performed on 510 camels, randomly selected from the 54 herds. The number of animals assessed in some herds, unfortunately, was lower than that one suggested, and this was due to logistic reasons (e.g., the animals had to move for grazing, and we did not want to cause further delay). Nevertheless, the total number of camels assessed is still a significant sample size of the total population. During the Animal level assessment, more than half of the camels had food available as they were grazing. It is worth noticing that the examined area, particularly the “Cholistan” desert, has a wide variety of vegetation that sustains the food needs of the camels (18). However, it is still a desert, so the scanty and rugged vegetation, coupled with the harsh environment, is uniquely suited to be utilized by camels, which are more resilient than other livestock. Except for brief wet periods, camels primarily depend on woody and bushy vegetation for sustenance (18). Hence, during dry periods, it’s crucial to have alternative food sources accessible, and district government and responsible bodies should educate pastoralists on methods for conserving food. Body condition score (BSC) was the other ABM of Good Feeding and confirmed the Caretaker-Herd level findings; over three-fourths of the evaluated camels were indeed classified as having good or moderate body condition, and less than a quarter were classified as cachexia or obese. During our study in the field, we encountered a lot of diverse wastes, including plastics, which could be ingested by the camels, leading to digestive issues and subsequently resulting in weight loss. Thus, it is crucial to emphasize waste management in the study localities, particularly concerning plastics, and caretakers should be educated about the potential hazards of foreign objects ingestion by camels. Another important indicator of Good Feeding is the availability of water. Water availability at the Animal level was very limited. This disagrees with what was reported by the caretakers, stating that they provide water more than once daily. During the assessment, there were few visible watering points for the camels in a few locations. Except for the observation of the surrounding environment, we had no better indicator for the water availability test, like the bucket test (9), which was not feasible, and there was a lack of particular indicators for prolonged thirst. In times of drought, the ponds containing water may dry up, leaving no water source for the camels. As a result, pastoralists often migrate to cultivated irrigated areas, causing conflicts on many occasions (18). In arid regions, access to drinking water for livestock is frequently restricted, as watering points are typically located several days’ journey away from grazing areas. Consequently, all livestock, especially during the dry season, routinely experience thirst (4). Even though, due to the anatomical and histological particularities of their kidneys, camels can withstand water deprivation (45), their ability to withstand water depends on many factors, including breed, climatic factors, quality and quantity of grazing vegetation, and its water content, purpose and the type of drought work they are involved in (46). Therefore, water should be available to camels, especially during the dry season and for the lactating camels, which require more water to produce milk, so that these camels do not suffer from prolonged thirst. Prolonged thirst could be a common welfare consequence for camels, mainly based on the belief that they do not need water, as they can survive without water longer than other animals. Similar findings have been reported, indeed, where camels were kept in a market in Doha, where the lack of a watering point was identified as a critical point that hindered the welfare of the camels (22). Since almost all the camels depended totally on grazing as a food source, and there was a critical lack of water in the area, it is not surprising that the Good Feeding PAI scored the lowest and was the most variable among the herds.

Under the assessment of Good Housing, the absence of shade was one of the most important critical points, where almost all assessed camels had no shade to prevent themselves from strong sun/ heat during the daytime. Zappaterra et al. (47) explored the preference of camels regarding shade. Despite the belief that camels, as desertic animals, do not need shade, the authors concluded that camels tend to prefer shaded areas during hot sunny days. Underscoring shade is, therefore, paramount in improving camels’ welfare. Animal welfare is indeed not only what animals need but also what animals want (48), so it is important to test their preference and provide what they prefer to ensure their welfare. As temperatures and humidity rise, livestock exhibit a preference for shade; the provision of shade significantly influences their behavior and contributes to enhancing their welfare while also aiding in minimizing thermal stress (49–52). On the other hand, most of the animals showed a clean coat and enjoyed freedom of movement. These animal-based measures compensated for the critical issues at the Caretaker-Herd level and could increase the final score of the Good Housing PAI. As mentioned before, however, it would be good if resting and sheltered points could be built along the common pastoralism ways so that camels could be watered, shaded, and rested not only at night but also when temperatures are above their thermal neutral zone (53).

Regarding Good Health, skin disorders, discharge, and injuries were mostly observed abnormalities and clinical signs in the examined camels during the animal-level assessment. However, it is worth noticing that despite these findings, most of the camels observed were so healthy that this welfare principle received one of the highest PAI scores. Ashraf et al. (54) reported trypanosomiasis, pneumonia, mange, and anthrax in the Cholistan desert, Raziq et al. (55) reported mange, Orf, camelpox, trypanosomosis, and contagious skin necrosis in Suleiman Mountainous Region in Pakistan, showing the extent of the incidence of camel disease in Pakistan. Our findings were expected as most of the clinical signs and abnormalities we reported are in line with those caused by the most common camel disease. However, it is worth noticing that it is not the scope of a welfare assessment to reach a diagnosis, so we do not know the real etiology of the clinical signs noticed. Throughout the globe, where camels are managed under nomadic pastoralists, semi-nomadic pastoralists, and semi-intensive systems, health problems are the main challenge in camel production, which is an obstacle to the welfare of these animals (32, 39, 56, 57). The pastoralist communities mostly move in search of food and water for their animals, and they tend to spend lots of time in remote locations where veterinary service is not easily accessible (18). Some of the disease conditions observed at the animal level could be of zoonotic importance, so awareness-creating training, including a detailed investigation of the diseases, should be conducted, and, as mentioned before, the veterinary service could be improved along the pastoralism ways. Another important indicator of good health is pain-inducing management procedures, which are practiced by pastoralists either to retrain or control the camels (nose rings/pag) (4) or as a traditional treatment method for different diseases (cauterization) (36). Despite overall good health, more than a quarter of our camels had experienced pain-induced management practices. These practices, either as control, restraining, or treatment procedures, should not cause prolonged pain to the camels. Restraining methods, like nose pag, are based on creating pain and discomfort, and they may affect the welfare of animals if used for prolonged periods (4).

As expected in camels kept under pastoralism, the behavioral needs were met, and the PAI for Appropriate Behavior was among the highest compared to the other PAIs. As expected, behavioral observations revealed minimal incidence of stereotypies and aggressive interactions in the examined animals. Oral and locomotory stereotypy were described in camel bulls kept in captivity when they were housed for 24 h in individual boxes. Restricting space allowance and limited social contact were revealed to be the causes of stereotypies in camels (58). Stereotypy frequency decreased when the bulls could be housed freely in a paddock and spend time close to females (59–61). Oral stereotypy was strongly dependent on the time of feeding in camels kept in intensive farming with rationed feeding regimes (59), so it is not surprising that our camels showed no oral stereotypy as they had all access to pasture and could meet their needs for grazing and browsing (62, 63). The observed camels showed many indicators of positive welfare, such as positive social interactions; many she-camels were seen taking care of their calves, and often camels were seen resting and ruminating close to each other, and some calves were also playing together. The fact that these animals live in a habitat closer to their natural one justifies their behavioral repertoire (63). This could also be the reason why, despite the belief that camels are aggressive animals, no aggressive interactions were recorded, and no aggressivity was reported as a behavioral problem by the caretakers. In the study conducted by Menchetti et al. (41), caretakers reported a higher incidence of behavioral problems in a camel market. However, in the latter study, camels were confined in their pens all day long with limited space in crowded conditions. The fact that our camels had the possibility to express their natural behavior and they were also managed gently by their owners reflected in the fact that the majority of them reacted neutrally or positively when a stranger tried to approach them, even though they were free to move and consequently to run away. Our findings may be useful in suggesting better standards for camels housed in intensive and semi-intensive systems.

The classifications of the herds were conducted using Principal Aggregate Indices (PAIs) and Total Welfare Index (TWI). Based on the PAI classification, none of the herds obtained an unacceptable level of classification, and four herds obtained excellent levels, indicating that the camels were managed well. These findings, moreover, suggest a better condition compared to intensively managed camels. Indeed, Menchetti et al. (22) have found in a camel market that no pens achieved the “excellent” category, and some pens had an “unacceptable” welfare level. Similarly, in intensively managed Dutch dairy cattle herds, “unacceptable” classification was also reported with no “excellent” classification (64). Based on the TWI, employing a traffic light classification, we were able to produce a threshold for this population (65.4 for the separation between Red and Orange light categories, 70.6 for the distinction between Orange and Green light categories). These thresholds were higher than those identified in intensive farming by Menchetti et al. (i.e., 56.0 and 62.0, respectively) (22). This confirms that camels managed under nomadic pastoralism in Pakistan have better welfare levels compared to intensively managed farm animals. The score of Appropriate Behavior PAI was the highest, probably contributing to the better result of herd classification compared to the previous study in the market. Animals managed under extensive systems tend to enjoy the expression of their natural behavior, have relative freedom (65), and have social contact with other camels and humans, making the animals more friendly (66). The critical areas that needed improvement were Good feeding, Good health, and Good housing. In particular, the Discriminant analysis showed that Good Feeding was the most influential variable in classifying and determining discrimination between different levels of welfare. These are expected results because continuity in food and water supply, shelter, and veterinary care are the major welfare concerns of all extensive systems (14, 67). To improve in these areas, particular attention should be given to the availability of water, the provision of shade, natural or manmade, to protect the camels from extreme heat, the availability of nighttime resting places and modern veterinary services to improve the well-being of the camels in the study localities.

Our data must be interpreted cautiously, as the study has several limitations. The initial limitation starts from the fact that the protocol was being applied for the first time and needs to be refined and validated by further studies. In particular, the validation of certain indicators within the pastoralist management production system is needed. The evaluation of the quality of the feed and water, when available, could not be also conducted. The presence of multiple individuals during the assessment in the open desert setting could potentially influence the outcome, as complete prevention of human presence was not possible. In addition, during observation the caretaker’s mood and techniques of handling the camels could be influenced due to the presence of the assessor in nearby distance, and could affect the outcome of the assessment. The other limitation is that in some of the herds, a limited number of animals were assessed due to logistic problems. Finally, it is important to highlight that this is a single welfare assessment conducted in a particular season (i.e., autumn), so the welfare classifications of the heard cannot be generalized for the full year, and multiple welfare assessments should be performed, as usually happen in every welfare scheme in other species. Notwithstanding these limitations, this study is the first to report the welfare levels of camels kept under pastoralism with an objective protocol. Our data may be useful to suggest recommendations tailored for the camels kept in Pakistan or similar conditions.

## 5 Conclusion

This study investigated the welfare of dromedary camels managed under a nomadic pastoral using a tailored protocol. The examined camels had better welfare compared to those in intensive systems, but critical welfare issues were identified. Key welfare risks included poor feeding, limited water availability, lack of shade, and insufficient overnight shelter. Health risks were also noted, with non-veterinarians treating camels and poor record-keeping. Stakeholders and the government should take action to address those issues. Our findings are a first step in proposing best practices for dromedary camels, not only in Pakistan but worldwide. They also are the groundwork for future research, providing valuable insight into the main welfare issues likely to be encountered in camels under the nomadic pastoral management system. Therefore, this work’s findings could help improve the welfare of the dromedary camels. The responsible stakeholders and government policymakers could benefit from this study’s findings in proposing and implementing appropriate policies and taking corrective action.

## Data Availability

The raw data supporting the conclusions of this article will be made available by the authors, without undue reservation.
