# Supplementary material for: Welfare assessment of dromedary camels kept under pastoralism in Pakistan
Source: Front Vet Sci. 2024 Oct 30;11:1442628. doi: 10.3389/fvets.2024.1442628 (PMC11557498; doi:10.3389/fvets.2024.1442628)
Supplement: Supplementary file 1 [file Table_1.docx]

Supplementary Material

**Welfare Assessment of Dromedary Camels Kept Under Pastoralism in Pakistan**

**Barbara Padalino^1,2^, Asim Faraz^3^, Naod Thomas Masebo^1,4*^, Abdul Waheed^3^, Hafiz**  **Muhammad Ishaq^3^, Nasir Ali Tauqir^5^, Ali Raza Abbasi^6^, Laura Menchetti^7^**

^1^Department of Agricultural and Food Sciences, University of Bologna, Italy.

^2^Faculty of Science and Engineering, Southern Cross University, Lismore, NSW, Australia,

^3^Department of Livestock and Poultry Production, Bahauddin Zakariya University Multan, Pakistan

^4^School of Veterinary Medicine, Wolaita Sodo University, Wolaita Sodo, Ethiopia

^5^Department of Animal Nutrition, The Islamia University of Bahawalpur, Pakistan

^6^Faculty of Veterinary and Animal Sciences, MNS University of Agriculture, Multan, Pakistan

^7^School of Biosciences and Veterinary Medicine, University of Camerino, Italy

*** Correspondence:**Naod Thomas Masebo

[naodthomas.masebo2@unibo.it](mailto:naodthomas.masebo2@unibo.it)

# Supplementary Figures and Tables

- 1. **Supplementary table**

**Supplementary Table 1:** Median, minimum, maximum and interquartile ranges (IQR) of partial indices (PIs) of fifty-four (*n*=54) Caretaker-Herd level assessments corresponding to the four welfare principles (i.e., Good feeding, Good housing, Good health and Appropriate Behavior).

| **Assessment level** | **Welfare principle** | **Partial Index (PI)** | **Min** | **Max** | **Median** | **Q1** | **Q3** | **IQR** |
| --- | --- | --- | --- | --- | --- | --- | --- | --- |
| **Caretaker-Herd** | Good Feeding | PI Good Feeding at Caretaker-Herd level | 25 | 75 | 50 | 50 | 50 | 0 |
|  | Good Housing | PI Good Housing at Caretaker-Herd level | 50 | 87.5 | 50 | 50 | 75 | 25 |
|  | Good Health | PI Good Health at Caretaker-Herd level | 35.7 | 71.4 | 42.9 | 42.9 | 42.9 | 0 |
|  | Appropriate Behavior | PI Appropriate Behavior at Caretaker-Herd level | 50 | 100 | 100 | 87.5 | 100 | 12.5 |

**Supplementary Table 2:** Median, minimum, maximum and interquartile ranges (IQR) of partial indices (PIs) of five hundred ten (*n*=510) Animal-level assessments corresponding to the four welfare principles (i.e., Good feeding, Good housing, Good health and Appropriate Behavior).

| **Assessment level** | **Welfare principle** | **Partial Index (PI)** | **Min** | **Max** | **Median** | **Q1** | **Q3** | **IQR** |
| --- | --- | --- | --- | --- | --- | --- | --- | --- |
| **Animal** | Good Feeding | PI Good Feeding at Animal level | 0 | 100 | 50 | 33 | 67 | 34 |
|  | Good Housing | PI Good Housing at Animal level | 25 | 94 | 75 | 62 | 75 | 13 |
|  | Good Health | PI Good Health at Animal level | 27 | 100 | 91 | 82 | 100 | 18 |
|  | Appropriate Behavior | PI Appropriate Behavior at Animal level | 10 | 100 | 80 | 60 | 90 | 30 |

**Supplementary Table 3:** The classification of each camel herd's (*n*=54) welfare status determined by the Principal Aggregate Indices (PAIs) in Pakistan in 2023. (excellent, satisfactory, unsatisfactory, unacceptable).

| **Herd ID** | **PAI Good Feeding** | **PAI Good Housing** | **PAI Good Health** | **PAI Appropriate Behavior** | **Welfare category** |
| --- | --- | --- | --- | --- | --- |
| 1 | 51.7 | 68.4 | 81.3 | 87 | Satisfactory |
| 2 | 40.6 | 68.3 | 71.6 | 70.7 | Satisfactory |
| 3 | 49.4 | 63.3 | 87 | 85.8 | Satisfactory |
| 4 | 45 | 62.5 | 84 | 71.1 | Satisfactory |
| 5 | 39.4 | 56.7 | 81.3 | 63.8 | Satisfactory |
| 6 | 50.2 | 57.9 | 80.8 | 76.6 | Satisfactory |
| 7 | 42.8 | 67.1 | 76.5 | 92.2 | Satisfactory |
| 8 | 46.5 | 51.1 | 88.6 | 70.1 | Satisfactory |
| 9 | 48.7 | 73 | 84.5 | 76.8 | Satisfactory |
| 10 | 47 | 71.4 | 86.1 | 77.6 | Satisfactory |
| 11 | 51.3 | 73 | 81.9 | 76.2 | Satisfactory |
| 12 | 45.6 | 71.7 | 79.8 | 60.3 | Satisfactory |
| 13 | 56.1 | 75.9 | 83.8 | 74 | Satisfactory |
| 14 | 54.4 | 76.7 | 81.3 | 86 | Satisfactory |
| 15 | 52.7 | 74 | 91.1 | 64.8 | Satisfactory |
| 16 | 24.7 | 69.5 | 76.4 | 69.4 | Unsatisfactory |
| 17 | 28.2 | 73.6 | 75.5 | 68.4 | Unsatisfactory |
| 18 | 33.3 | 70 | 88.6 | 76.8 | Satisfactory |
| 19 | 32.2 | 70 | 78.9 | 82.3 | Satisfactory |
| 20 | 34.4 | 70 | 82 | 81.8 | Satisfactory |
| 21 | 23.3 | 70 | 77.7 | 80 | Unsatisfactory |
| 22 | 26 | 70 | 82.6 | 86.4 | Unsatisfactory |
| 23 | 24.7 | 69 | 81 | 82.3 | Unsatisfactory |
| 24 | 31 | 81.1 | 86.9 | 78.3 | Satisfactory |
| 25 | 23.3 | 70 | 82.5 | 79.2 | Unsatisfactory |
| 26 | 28.7 | 70 | 82.5 | 82.4 | Unsatisfactory |
| 27 | 40.6 | 62.1 | 72.2 | 82.3 | Satisfactory |
| 28 | 49 | 56 | 76.5 | 79.2 | Satisfactory |
| 29 | 52 | 72.8 | 82.9 | 71.2 | Satisfactory |
| 30 | 62.4 | 68.3 | 86 | 72.8 | Satisfactory |
| 31 | 58 | 72.2 | 84.4 | 87.6 | Satisfactory |
| 32 | 62.6 | 74.3 | 83.8 | 84.9 | Excellent |
| 33 | 53.3 | 77.5 | 82.2 | 77 | Satisfactory |
| 34 | 53.7 | 71.5 | 83.4 | 73.4 | Satisfactory |
| 35 | 44.3 | 76.4 | 80.1 | 78.3 | Satisfactory |
| 36 | 52.3 | 61 | 73.5 | 76 | Satisfactory |
| 37 | 53.3 | 70 | 81.3 | 78 | Satisfactory |
| 38 | 45.6 | 61.7 | 76.5 | 73.3 | Satisfactory |
| 39 | 36.7 | 53.8 | 56.7 | 75.7 | Satisfactory |
| 40 | 29 | 64.6 | 78 | 65.6 | Unsatisfactory |
| 41 | 74.6 | 71.2 | 81.3 | 93.8 | Excellent |
| 43 | 84 | 69.1 | 85.8 | 89.9 | Excellent |
| 44 | 72.6 | 65.4 | 81.3 | 75 | Satisfactory |
| 45 | 73.7 | 62.8 | 77.1 | 87 | Excellent |
| 46 | 38.7 | 71.2 | 80.7 | 86.7 | Satisfactory |
| 47 | 35.6 | 57 | 73.9 | 82 | Satisfactory |
| 48 | 45 | 53.1 | 71.1 | 81 | Satisfactory |
| 49 | 50 | 57.1 | 65.1 | 79.4 | Satisfactory |
| 50 | 51.3 | 58 | 79.4 | 92.8 | Satisfactory |
| 51 | 81.1 | 65.2 | 70.3 | 74.9 | Satisfactory |
| 52 | 78.7 | 63 | 80.8 | 74.4 | Satisfactory |
| 53 | 58.8 | 64.6 | 81.2 | 87.4 | Satisfactory |
| 54 | 50 | 66.9 | 78.9 | 86.3 | Satisfactory |
| 55 | 50.8 | 60.6 | 79.9 | 69.9 | Satisfactory |

**Supplementary Table 4:** The classification of the welfare category of each camel herd (*n*=54) based on the total welfare indices (TWIs) in Pakistan in 2023.

| **Herd ID** | **TWI** | **Welfare category** |
| --- | --- | --- |
| 1 | 72.1 | Green light |
| 2 | 62.8 | Red light |
| 3 | 71.4 | Green light |
| 4 | 65.7 | Orange light |
| 5 | 60.3 | Red light |
| 6 | 66.4 | Orange light |
| 7 | 69.6 | Orange light |
| 8 | 64.1 | Red light |
| 9 | 70.7 | Green light |
| 10 | 70.5 | Orange light |
| 11 | 70.6 | Orange light |
| 12 | 64.3 | Red light |
| 13 | 72.4 | Green light |
| 14 | 74.6 | Green light |
| 15 | 70.6 | Green light |
| 16 | 59.9 | Red light |
| 17 | 61.4 | Red light |
| 18 | 67.2 | Orange light |
| 19 | 65.9 | Orange light |
| 20 | 67.1 | Orange light |
| 21 | 62.7 | Red light |
| 22 | 66.3 | Orange light |
| 23 | 64.2 | Red light |
| 24 | 69.3 | Orange light |
| 25 | 63.8 | Red light |
| 26 | 65.9 | Orange light |
| 27 | 64.3 | Red light |
| 28 | 65.2 | Red light |
| 29 | 69.7 | Orange light |
| 30 | 72.4 | Green light |
| 31 | 75.5 | Green light |
| 32 | 76.4 | Green light |
| 33 | 72.5 | Green light |
| 34 | 70.5 | Orange light |
| 35 | 69.8 | Orange light |
| 36 | 65.7 | Orange light |
| 37 | 70.7 | Green light |
| 38 | 64.3 | Red light |
| 39 | 55.7 | Red light |
| 40 | 59.3 | Red light |
| 41 | 80.2 | Green light |
| 43 | 82.2 | Green light |
| 44 | 73.6 | Green light |
| 45 | 75.1 | Green light |
| 46 | 69.3 | Orange light |
| 47 | 62.1 | Red light |
| 48 | 62.6 | Red light |
| 49 | 62.9 | Red light |
| 50 | 70.4 | Orange light |
| 51 | 72.9 | Green light |
| 52 | 74.2 | Green light |
| 53 | 73.0 | Green light |
| 54 | 70.5 | Orange light |
|  | 65.3 | Red light |

## Supplementary Table 5: Functions at Group Centroids of the Discriminant Analyses investigating the classification of the "welfare profiles system” and “light traffic system”.

| **Classification system** | | | | | | |
| --- | --- | --- | --- | --- | --- | --- |
| **Welfare profiles** | | |  | **Light traffic** | | |
| **Category** | **Function** | |  | **Category** | **Function** | |
|  | **1** | **2** |  |  | **1** | **2** |
| **Excellent** | 2.995 | 0.768 |  | **Green light** | 2.263 | -0.313 |
| **Satisfactory** | 0.128 | -0.165 |  | **Orange light** | 0.003 | 0.624 |
| **Unsatisfactory** | -2.168 | 0.480 |  | **Red light** | -2.266 | -0.311 |
| Unstandardized canonical discriminant functions evaluated at group means | | | | | | |

## Supplementary figures


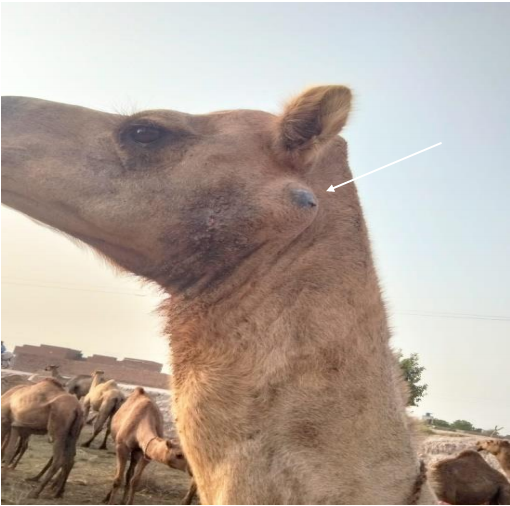


**Supplementary Figure 1**: Examples of camels not included in the welfare assessment due to health conditions: An absecces (indicated in the arrow).


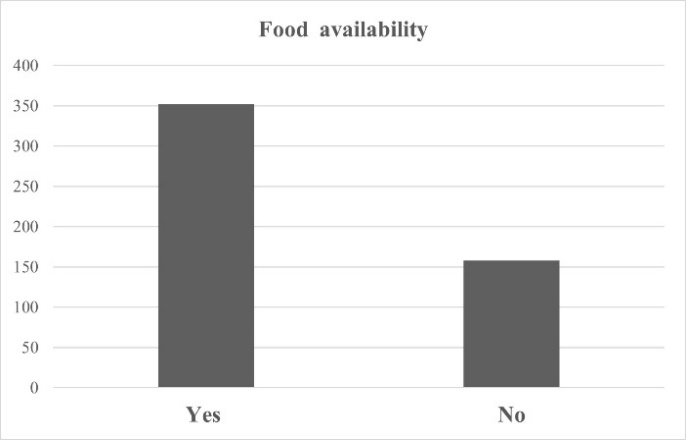

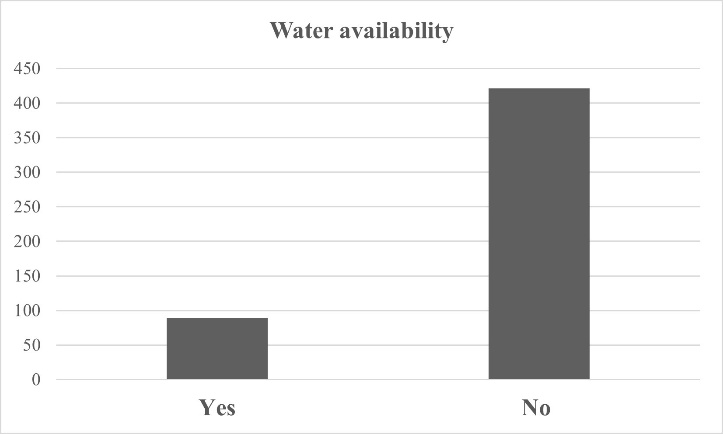


(A) (B)


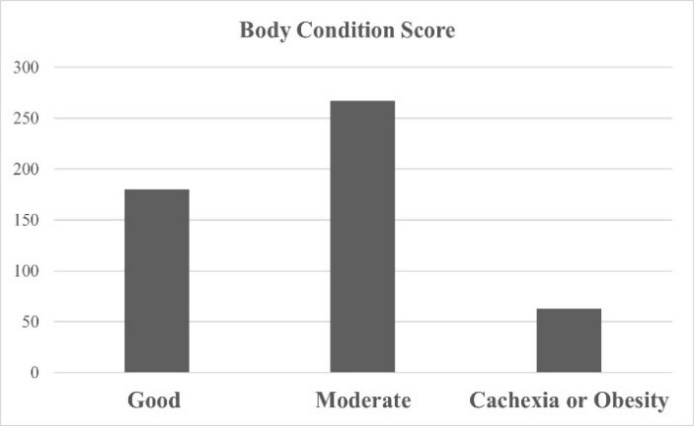

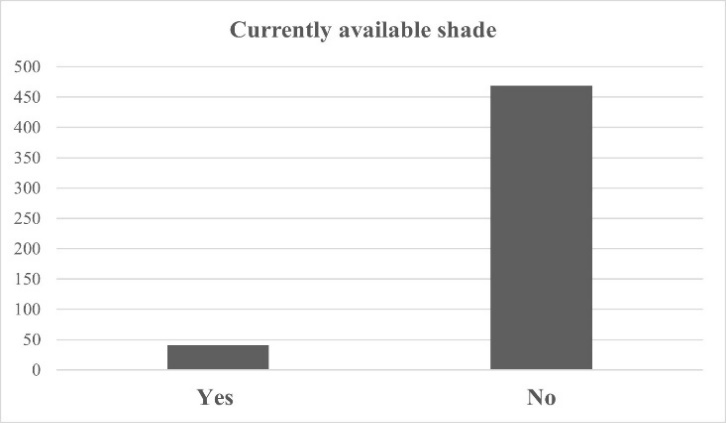


(C) (D)


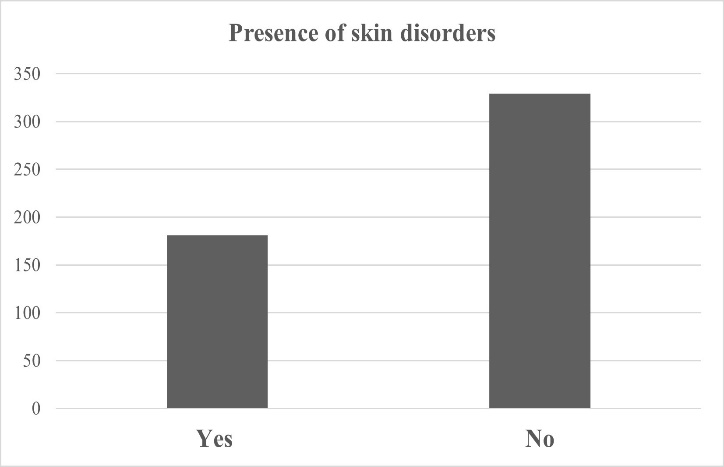

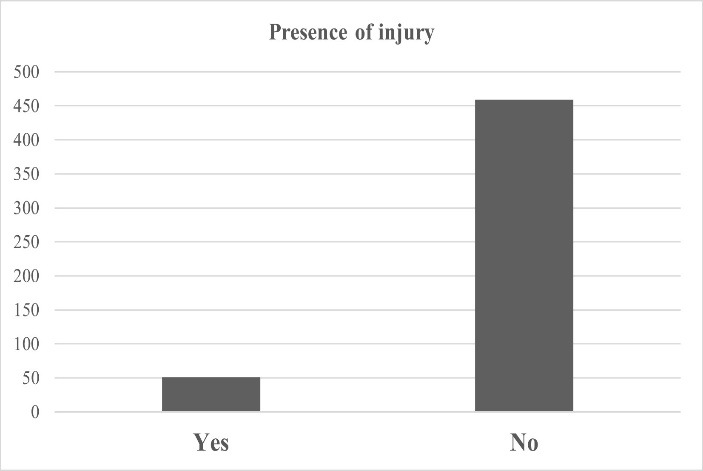


(E) (F)


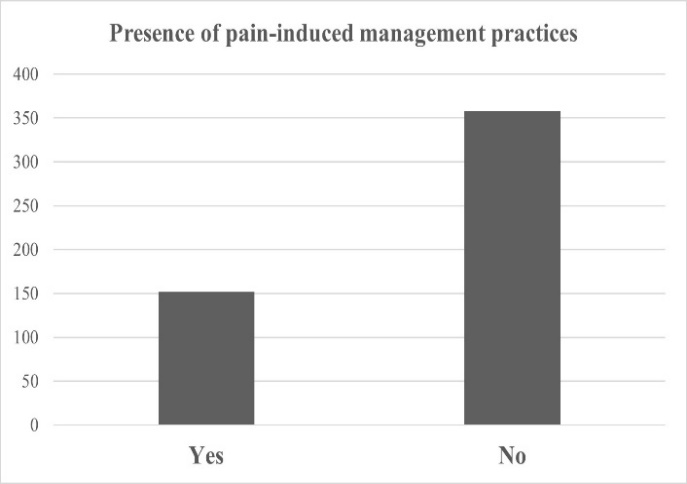

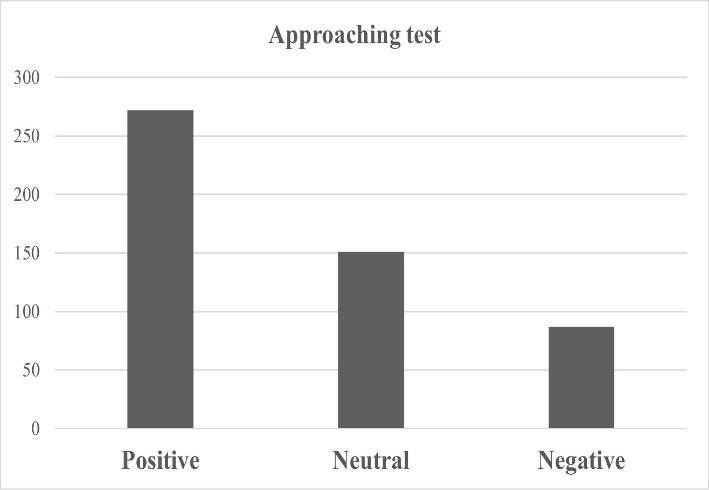


(G) (H)

**Supplementary Figure 2**: Distribution of selected animal-level assessment indicators (A) Food Availability, (B) Water Availability, (C) Body condition score, (D) Currently Available shade, (E) Presence of skin disorder, (F) Presence of injury, (G) Presence of pain-induced management practices, (H) Approaching test

**Supplementary Figure 3.** Boxplot for partial indices (PIs) at Caretaker-Herd (C-H) and Animal levels corresponding to the four welfare principles (i.e., Good feeding, Good housing, Good health, and Appropriate Behavior). The whiskers on the plot define the range from the 2.5 to the 97.5 percentile while the dots show the outliers (that fall below or above this range).
